# Spatial and Temporal Control of 3D Hydrogel Viscoelasticity through Phototuning

**DOI:** 10.1021/acsbiomaterials.3c01099

**Published:** 2023-11-29

**Authors:** Philip Crandell, Ryan Stowers

**Affiliations:** † Department of Mechanical Engineering, University of California, Santa Barbara, Santa Barbara, California 93016, United States; ‡ Biological Engineering Program, University of California, Santa Barbara, Santa Barbara, California 93106, United States

## Abstract

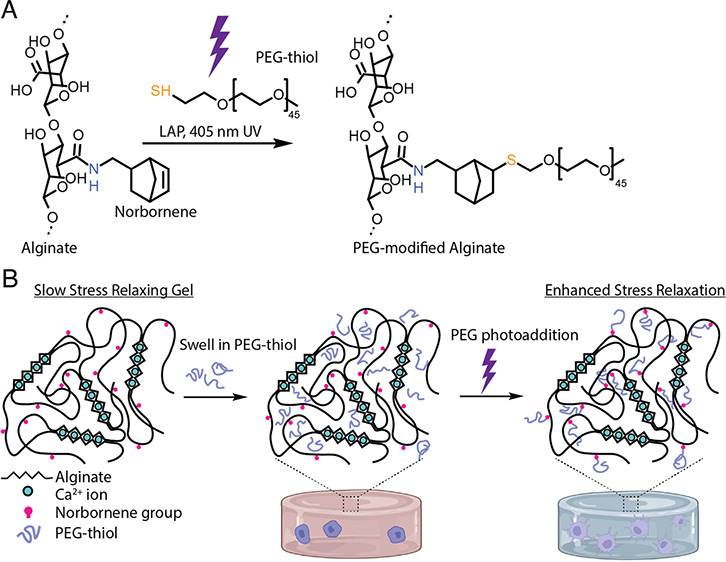

The mechanical properties of the extracellular environment can regulate a variety of cellular functions, such as spreading, migration, proliferation, and even differentiation and phenotypic determination. Much effort has been directed at understanding the effects of the extracellular matrix (ECM) elastic modulus and, more recently, stress relaxation on cellular processes. In physiological contexts such as development, wound healing, and fibrotic disease progression, ECM mechanical properties change substantially over time or space. Dynamically tunable hydrogel platforms have been developed to spatiotemporally modulate a gel’s elastic modulus. However, dynamically altering the stress relaxation rate of a hydrogel remains a challenge. Here, we present a strategy to tune hydrogel stress relaxation rates in time or space using a light-triggered tethering of poly(ethylene glycol) to alginate. We show that the stress relaxation rate can be tuned without altering the elastic modulus of the hydrogel. We found that cells are capable of sensing and responding to dynamic stress relaxation rate changes, both morphologically and through differences in proliferation rates. We also exploited the light-based technique to generate spatial patterns of stress relaxation rates in 3D hydrogels. We anticipate that user-directed control of the 3D hydrogel stress relaxation rate will be a powerful tool that enables studies that mimic dynamic ECM contexts or as a means to guide cell fate in space and time for tissue engineering applications.

## Introduction

Hydrogels are widely used to simulate aspects of the native extracellular matrix (ECM) for in vitro cell culture.^1^ Key biophysical and biochemical properties of the ECM can be precisely controlled in hydrogel cultures, enabling reductionist investigations into the effect of the cellular microenvironment or design of artificial ECMs for in vitro cell and tissue models.^2^ The mechanical properties of the hydrogel network are particularly important in regulating cell behaviors such as cell proliferation, adhesion, migration, and spreading. Indeed, the stiffness (elastic modulus) of the hydrogel can direct differentiation of stem cells, enhance cancer cell malignant traits, enable or restrict vascularization, and control organoid formation.^3−8^ While the importance of mimicking or controlling the elastic modulus of a hydrogel matrix is now well-recognized, most soft tissues in the body do not behave purely elastically, but instead exhibit properties of both elastic solids and viscous fluids.^9,10^ The viscous component of the tissues dissipates energy over time, resulting in time-dependent mechanical phenomena, such as stress relaxation (a decrease in stress under a constant strain) or creep (an increase in strain under constant stress). Recently, differences in matrix viscoelasticity have been shown to impact cell fate and phenotype as well, underscoring the importance of mimicking this mechanical behavior in in vitro models of the cellular microenvironment.^11−16^

Many widely used hydrogel cross-linking chemistries form irreversible covalent bonds, and the resulting hydrogels behave elastically; that is, stress is stored indefinitely upon deformation, not dissipated over time. In order to generate highly viscoelastic gels, the polymer network must be able to be rearranged in response to force through disruption of the network cross-links. Several strategies have been employed toward this end, including the use of ionic cross-links, guest–host and other supramolecular interactions, dynamic covalent bonds, and hydrophobic interactions.^11,13,17,18^ These hydrogel systems have been used to interrogate the cellular response to viscoelasticity, a burgeoning area in mechanobiology and 3D culture models. Mesenchymal stem cell spreading, focal adhesion formation, and osteogenic differentiation are significantly enhanced in fast-relaxing matrices.^11−14^ Viscoelastic matrices have been used to generate, expand, and passage stem cell-derived organoids, and the extent of local viscoelasticity can enable morphogenetic processes such as intestinal organoid budding.^16,19−21^ The tumor microenvironment is also viscoelastic, and cancer cells are responsive to different rates of stress relaxation through different rates of proliferation, migration, and modes of invasion.^22−25^

**Figure 1:**
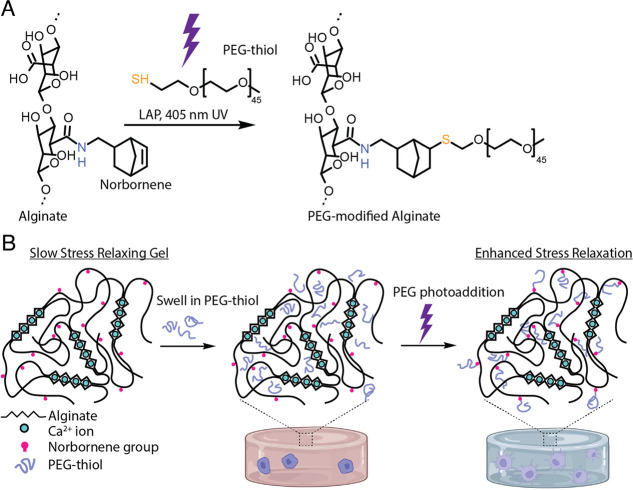
Strategy to generate light-triggered changes in the alginate hydrogel stress relaxation rate. (a) Chemical structures of alginate modified with norbornene and thiol–ene reactions to conjugate PEG to alginate in the presence of 405 nm light and the photoinitiator lithium phenyl-2,4,6-trimethylbenzoylphosphinate (LAP). (b) Schematic of the alginate polymer network during photoaddition of PEG chains that serve to enhance the stress relaxation rate of the gel.

Tissue mechanical properties vary throughout time during many biological processes, such as development and aging, and diseases such as organ fibrosis and solid tumor progression.^26−30^ Similarly, tissues are spatially heterogeneous in terms of mechanical properties.^9,31^ Dynamically tunable hydrogels have been developed to mimic the spatial and temporal changes that occur in tissues.^32−40^ These approaches primarily rely on methods to alter hydrogel cross-link density in a temporal or spatial gradient or with a user-directed stimulus like light, heat, or ultrasound. Cross-link density is directly related to the hydrogel network elasticity; thus, the stiffness of these gels can be altered spatiotemporally. It was recently shown that hyaluronic acid hydrogels could be dynamically stiffened while maintaining viscoelasticity by using light-triggered incorporation of secondary guest–host bonds.^17^ However, altering the viscoelasticity of a hydrogel in time or space, for example, the rate at which stress relaxes under constant strain without simultaneously altering the stiffness, remains a challenge. Very recently, this challenge was overcome using PEG gels and a method that enables light-triggered exchange of cross-link bonds to permit stress relaxation, enabling control of viscoelasticity.^21,41^ Here, we demonstrate a strategy to spatiotemporally modulate 3D alginate hydrogel stress relaxation without altering stiffness and in the presence of cells. Our platform utilizes alginate gels cross-linked with calcium, which are inherently viscoelastic due to the nature of the ionic bonds. Modification of alginate with monofunctional PEG chains can enhance the stress relaxation rate of the gels in a concentration-dependent manner.^42^ Our approach is to employ photoclick chemistry to conjugate PEG chains to an existing, 3D, cell-laden alginate network to modify its viscoelasticity without altering the stiffness of the gel (Figure 1).

## Materials and Methods

### Alginate Preparation

Alginate (280 kDa molecular weight, LF 20/40) from the FMC biopolymer was dissolved at 1% in deionized water and dialyzed with 10 kDa MWCO membranes against deionized water for 3 days. Following dialysis, alginate was purified with activated charcoal, sterile-filtered, frozen, and lyophilized.

### Functionalization of Alginate with RGD

RGD peptides were coupled to alginate by using carbodiimide chemistry. Alginate was dissolved in 0.1 M 2-(*N*-morpholino)ethanesulfonic acid (MES) buffer at pH 6.5. Then, appropriate amounts of sulfo-NHS (*N*-hydroxysulfosuccinimide), *N*-(3-(dimethylamino)propyl)-*N*′-ethyl carbodiimide hydrochloride (EDC), and the peptide sequence GGGGRGDSP were mixed and the reaction was left to stir for 20 h at room temperature.^14,43^ The product was then transferred to 10 kDa MWCO dialysis tubing and dialyzed against decreasing concentrations of NaCl solutions starting from 120 to 0 mM over 2 days, followed by 1 day of dialysis against deionized water. Water was then removed via lyophilization to yield functionalized alginate.

### Functionalization of Alginate with Norbornene

Alginate functionalized with RGD was additionally functionalized with norbornene using a procedure similar to the above. Alginate-RGD was dissolved in MES buffer and functionalized with sulfo-NHS, EDC, and norbornene (5-norbornene-2-methylamine, TCI Chemicals). After sulfo-NHS, EDC, and alginate-RGD were allowed to dissolve, the pH of the MES was raised to 8, and norbornene was added. Following the reaction, alginate was dialyzed and lyophilized as described above. Lyophilized alginate was dissolved in phenol red-free Dulbecco’s Modified Eagle Medium (DMEM) at 3% M/V. Free norbornenes were quantified by reacting with 2 kDa mPEG-thiols and Ellman’s reagent (5,5-dithio-bis(2-nitrobenzoic acid)). The remaining thiols were quantified with Ellman’s reagent to determine the norbornene substitution of alginate.

### Hydrogel Formation and Tuning of Mechanical Properties

To tune the viscoelasticity of alginate after gelation, 2 kDa mPEG-thiol chains were reacted with norbornene groups on alginate. Alginate gels were formed, as described previously.^44^ Briefly, a syringe containing alginate was coupled to a second syringe containing calcium sulfate in DMEM, and the contents of both syringes were rapidly mixed. Hydrogels were cast directly into 8-well chambered cover glasses or cast between two silanized glass plates spaced 2 mm apart and punched into 8 mm diameter disks. After gelation for 40 min at 37 °C, gels were equilibrated in DMEM. For photocoupling, PEG-thiol and LAP (lithium phenyl-2,4,6-trimethylbenzoylphosphinate) in phenol red-free DMEM were added to the well either immediately postgelation or after 24 or 72 h. PEG was allowed to swell in for 4 h, then gels were exposed to 60 s of 405 nm light, and the media was changed to remove any unreacted PEG.

### Mechanical Characterization

Hydrogel mechanical properties were characterized on a TA Instruments ARES G2 strain-controlled rheometer with 8 mm parallel plates. Alginate hydrogels with and without PEG were formed, as described above. All hydrogel samples were measured 24 h after photoaddition of PEG, if applicable. The top plate was brought down until it registered a non-negative axial force, and the gap between plates was filled with DMEM. Shear modulus was measured using a frequency sweep from 0.1 to 10 Hz at a strain of 1%. The elastic modulus (*E*) was calculated assuming a Poisson’s ratio (ν) of 0.5 using eq 1:
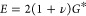
1where the complex modulus (G*) was determined from the storage modulus (*G*′) and loss modulus (*G*′′) using eq 2:
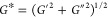
2

For stress relaxation tests, a constant strain of 15% was applied, and stress was recorded over time. Relaxation time was defined as the time taken for the stress to relax to half of its initial value. For creep tests, a constant 100 Pa stress was applied to each alginate gel for 3600 s, and the gel was allowed to recover at 0 Pa applied stress for 7200 s. Control values for each creep-recovery test were derived via a frequency sweep performed directly before the test.

### Cell Culture

Mouse D1 MSCs (ATCC, CRL-12424) were cultured in DMEM with 10% fetal bovine serum and 1% penicillin/streptomycin. Mouse D1 cells were passaged at approximately 50% confluency, and the media was changed every 48 h. MDA-MB-231 cells (ATCC, HTB-26) were expanded in DMEM containing 10% fetal bovine serum and 1% penicillin/streptomycin. The medium was changed every 48 h, and the cells were passaged at 70% confluency.

### Staining and Microscopy

To evaluate the cell morphology, cells were grown in hydrogels cast into a chambered cover glass (Nunc Lab-Tek II). After the last day of the culture period, cells were fixed using 4% paraformaldehyde in serum-free DMEM at 37 °C for 1 h. Gels were then washed 3 times in PBS containing calcium and 0.1% Triton X-100 for 30 min each. Alexa Fluor 555 phalloidin and DAPI were added for 90 min at room temperature, and the sample was again washed 3 times with calcium PBS at 30 min intervals. Samples were imaged immediately by using a Leica SP8 laser scanning confocal microscope with a 25× magnification water immersion objective.

### Proliferation Assay

To quantify proliferation, cells were encapsulated in hydrogels as described above, and media containing 10 μM 5-ethynyl-2′-deoxyuridine (EdU) was added 24 h before fixation. Gels were fixed in 4% paraformaldehyde for 45 min, washed 3 times with PBS containing calcium, and incubated with 30% w/v sucrose in calcium-containing PBS overnight. Gels were then placed in a mixture of 50% sucrose and 50% Tissue Tek O.C.T. compound on a shaker for 8 h before being frozen in O.C.T. and sectioned. Sectioned gels were stained for EdU using a 647 fluorescent EdU kit (Click-and-Go EdU 647, Click Chemistry tools) per the manufacturer’s directions. After functionalizing EdU with fluorophore, sections were incubated in 1:1000 DAPI for 30 min and washed 3x with PBS.

### Photopatterning of Alginate

Patterned photomasks were produced using a laser printer to transfer toner to an 8 × 10 in. polystyrene sheet (Shrinky Dink). Sheets were then cut and placed in an oven at 160 °C for 2 min, similar to previous methods.^45^ A glass slide was placed on the sheet as it shrunk to ensure that the pattern remained flat. The patterns were transferred to the gels in a chambered coverglass using a collimated 405 nm laser (NDV4512, Laserlands) by placing the photomask against the glass surface of the sample and illuminating through it for 30 s.

### Image Analysis

All images were collected using a Leica SP8 confocal microscope with a 0.95 NA 25× magnification water immersion objective. Metrics describing cell morphology in three dimensions, such as sphericity and volume, were quantified using Bitplane Imaris 9.5 software. In Imaris, sphericity is defined as the ratio of the surface area of a sphere with the same volume as the cell to the surface area of the cell itself. Solidity of a maximum projection of a 3D stack was quantified using ImageJ, where the solidity represented the difference between the convex hull area and the area of the cell itself. All 2D images of cell proliferation staining were analyzed and quantified by counting the number of costained DAPI and EdU cells and taking that as a fraction out of each separate field of view. Analysis of morphology in patterned gels was performed on a single stitched stack from 3 technical replicates, each 50 μm deep and with a 3 mm^2^ area.

### Statistical Analysis

Statistical comparisons were performed using GraphPad Prism 9.5. One-way analysis of variance was used to compare more than two groups. For measurements like cell volume, sphericity, and solidity, the D’Agostino-Pearson normality test was first performed to test if the data could be treated normally. For cell morphology experiments, approximately 25 cells were collected per trial, and data from 3 separate trials were pooled for analysis. A total of 18 fields of view were analyzed from 2 separate trials in each condition. Image analysis of patterned gels was performed on a single stitched stack from 3 technical replicates. Cells from photopatterned gels were analyzed using 2D metrics because the vertical sampling rate was insufficient for 3D analysis, and values for circularity, roundness, and solidity were reported.

## Results and Discussion

### Photocoupling of PEG to 3D Alginate Hydrogel Networks Enhances Stress Relaxation Rate

First, we modified high-molecular-weight alginate, previously shown to have slow stress relaxation, with norbornene functional groups to enable photoclick conjugation of monofunctional PEG-thiol chains.^11^ Norbornene methylamine was grafted to alginate using carbodiimide chemistry, a well-established chemistry^46^ for functionalizing amines to alginate’s carboxylic acid groups (Figure 1A). Quantification of this reaction using Ellman’s reagent found that 7% of the carboxylic acids was substituted with norbornenes. Norbornene functional groups can react with free thiols in solution in the presence of a photoinitiator via the cytocompatible thiol–ene reaction (Figure 1A). Alginate modified with PEG chains before gelation has been shown to have increased stress relaxation compared to unmodified alginate, and the stress relaxation rate increases with the amount of PEG added.^42,44^ Here, we demonstrate the extent to which stress relaxation can be tuned by photocoupling of PEGs in a preformed 3D gel and in the presence of cells (Figure 1B).

**Figure 2:**
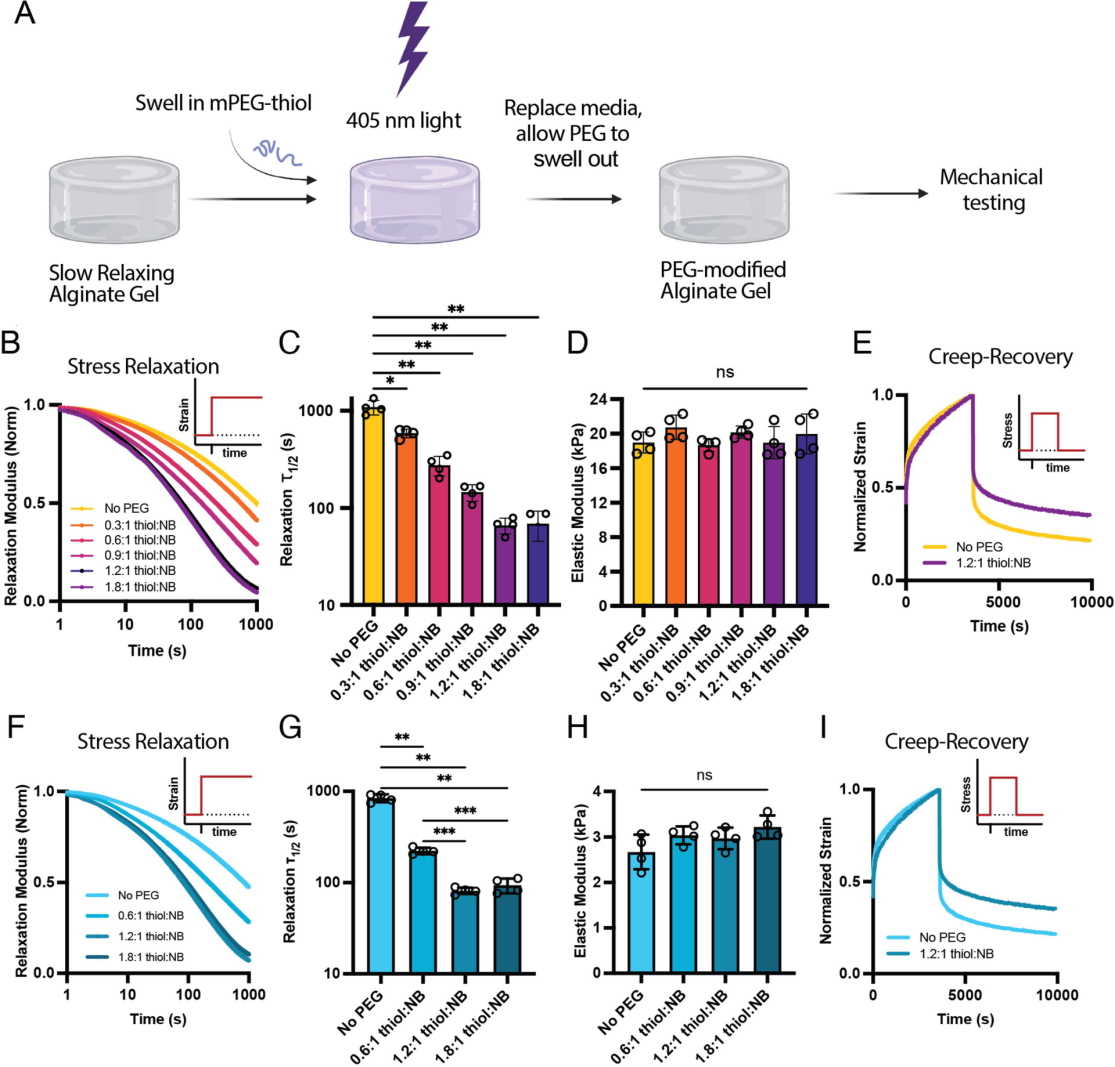
Light-triggered changes to alginate hydrogel viscoelasticity. (a) Schematic of the experimental timeline. Norbornene-modified alginate hydrogels were equilibrated with a solution of mPEG-SH and LAP, exposed to 405 nm light, and unreacted PEG was allowed to diffuse out of the hydrogel before mechanical testing. Hydrogels were initially either 20 kPa in elastic modulus (b–e) or 3 kPa (f–i). (b, c, f, g) Stress relaxation rates can be enhanced by incorporating mPEG into the alginate network, with a dependency on the amount of PEG added. (d, h) PEG photoconjugation does not significantly alter the hydrogel elastic modulus. (e, i) Creep-recovery tests demonstrate that PEG additional also alters the irrecoverable viscous deformation of the hydrogel network.

**Figure 3:**
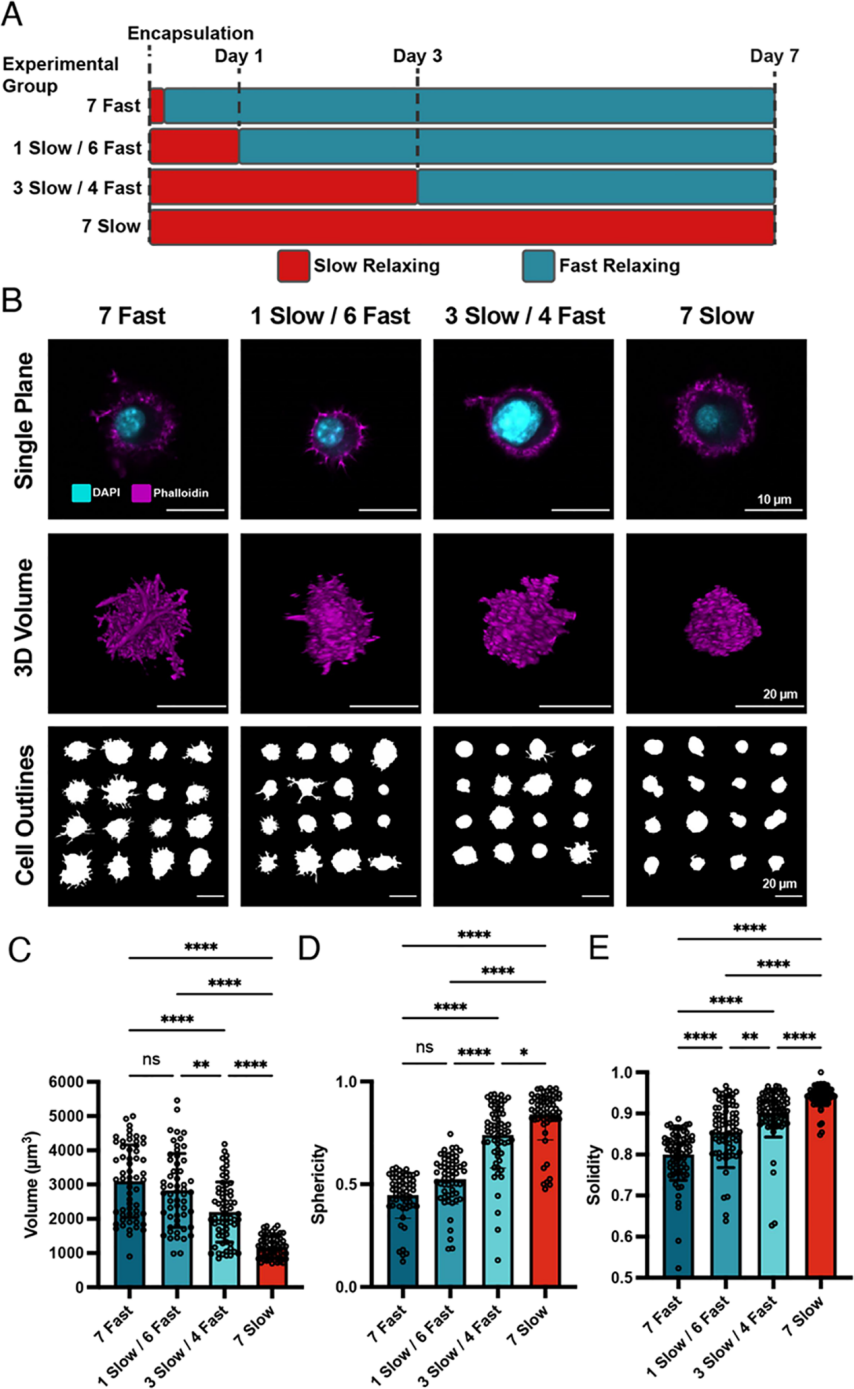
Cell spreading is responsive to dynamic viscoelasticity changes. (a) Experimental timeline to evaluate cellular responses to changes in viscoelasticity on days 0, 1, 3, or no changes (slow-relaxing control). Cells were analyzed on day 7 for all conditions. (b) MSCs exhibit spread morphologies when hydrogel stress relaxation is dynamically enhanced. Cross-sectional images of single MSCs, volumetric reconstructions of *z*-stacks, and outlines of maximum projections are shown. The cell outlines illustrate a representative sample of the distribution of cell shapes observed in our data set. (c–e) Quantification of shape metrics (volume (c), sphericity (d), and solidity (e)) reveal significantly more spread and protrusive shapes as cells spend more time in fast-relaxing hydrogels.

To characterize the mechanical changes in alginate gels after PEG-addition in conditions that mimic cell culture situations, norbornene-alginate hydrogels were ionically cross-linked and cut into 8 mm cylindrical samples with a biopsy punch to ensure samples had the same initial mechanical properties. To modify mechanical properties after gelation, gels were equilibrated with varying concentrations of monofunctional 2 kDa PEG-thiol and the photoinitiator LAP, exposed to a 405 nm laser, then swollen in buffer to remove any unreacted PEG chains (Figure 2A). Gels were made to have an initial elastic modulus of either 20 (Figure 2B–E) or 3 kPa (Figure 2F–I). Alginate hydrogels with more photocoupled PEG, presented as the ratio of PEG-thiols to norbornenes, produced faster-relaxing hydrogels, demonstrating the proof-of-concept of our approach (Figure 2B,C,F,G). For ease of comparison of stress relaxation rates, we determined the time necessary to reach half of the initial stress (τ_1/2_). Relaxation half-times varied from 840 s for unmodified alginate to 82 s for hydrogels where the concentration of added thiol exceeded the number for norbornene groups. Adding PEG in a 1.8 molar excess to norbornene produced similar stress relaxation times to a 1.2 molar excess, indicating that there is a diminishing effect at high PEG concentrations. Importantly, the elastic moduli were not significantly affected by PEG photoaddition, demonstrating that these two mechanical parameters can be independently modulated (Figure 2D,H). Creep-recovery tests were performed on unmodified and 1.2:1 thiol: norbornene hydrogels. In a creep-recovery test, strain is measured, while a constant stress is applied to the gel (creep), followed by a period of zero stress (recovery). PEG-modified hydrogels had significantly higher residual strains after creep-recovery testing, indicating more plastic deformation in these gels (Figure 2E,I).

Together, these results show that photoaddition of PEGs in alginate hydrogels can allow for on-demand changes in the viscoelastic behavior of alginate hydrogels, independent of the elastic modulus. Importantly, this range of stress relaxation rate, from approximately 100–1000 s, spans the measured relaxation rates for many soft tissues^10^ and the spectrum over which cellular behaviors are drastically altered.^11,14,42^ This range of stress relaxation rates compares favorably to most engineered hydrogel systems, though it does not relax as rapidly as the fastest relaxing tissues with τ_1/2_ on the order of 10 s. It is possible that more rapid relaxation could be achieved by modifying alginate to a greater extent with norbornene. A unique feature of this method is that the mechanism of dynamically modulating the stress relaxation does not affect the hydrogel cross-link density, and thus hydrogel stiffness is not altered by stress relaxation changes. While this platform represents an advance in dynamically tunable hydrogel platforms, we note two limitations in our approach. First, stress relaxation can be modulated in only one direction, from slow relaxing to fast relaxing. Second, PEG chains must be swollen into the hydrogel network, which limits the time over which modulation can be performed to several hours. We will seek to overcome these challenges in future iterations of this engineered platform.

### Cell Spreading is Promoted by Dynamically Enhanced Stress Relaxation

After establishing our platform for phototunable viscoelastic properties, we then sought to determine how cells respond to dynamic changes in stress relaxation rates. Alginate does not possess binding sites for cell adhesion, so peptides presenting the RGD-adhesion motif were coupled to norbornene-alginate to allow for cell adhesion.^11^ Prior reports have demonstrated that mesenchymal stem cells (MSCs) have increasing protrusions and spreading in fast-relaxing matrices compared to rounded morphologies in slow-relaxing matrices.^11^ We sought to determine if cell spreading could be induced on demand by transitioning from slow-relaxing to fast-relaxing conditions in the presence of cells (Figure 3A). The amount of time for slow-relaxing conditions before PEG conjugation was varied for encapsulated MSCs. As expected, cells cultured in slow-relaxing matrices for 7 days were highly spherical with few protrusions (Figure 3B). Intriguingly, cells in matrices that were transitioned from slow relaxing to fast relaxing were significantly less spherical, had significantly larger volumes, and had more protrusions, captured by the solidity metric (Figure 3B–E). These results show that the range of viscoelastic tunability of our approach is sufficient to observe differences in cellular response and that cells can respond to temporal changes to viscoelasticity. Interestingly, the magnitude of cell shape differences depended on the time of transition, with a diminished effect for cells transitioning later in the 7-day culture period (i.e., 3 days in slow-relaxing matrices/4 days in fast-relaxing matrices). Since both the time the cells were cultured in slow-relaxing conditions and fast-relaxing conditions were varied in this experimental design, the effects of each gel condition could not be decoupled.

**Figure 4:**
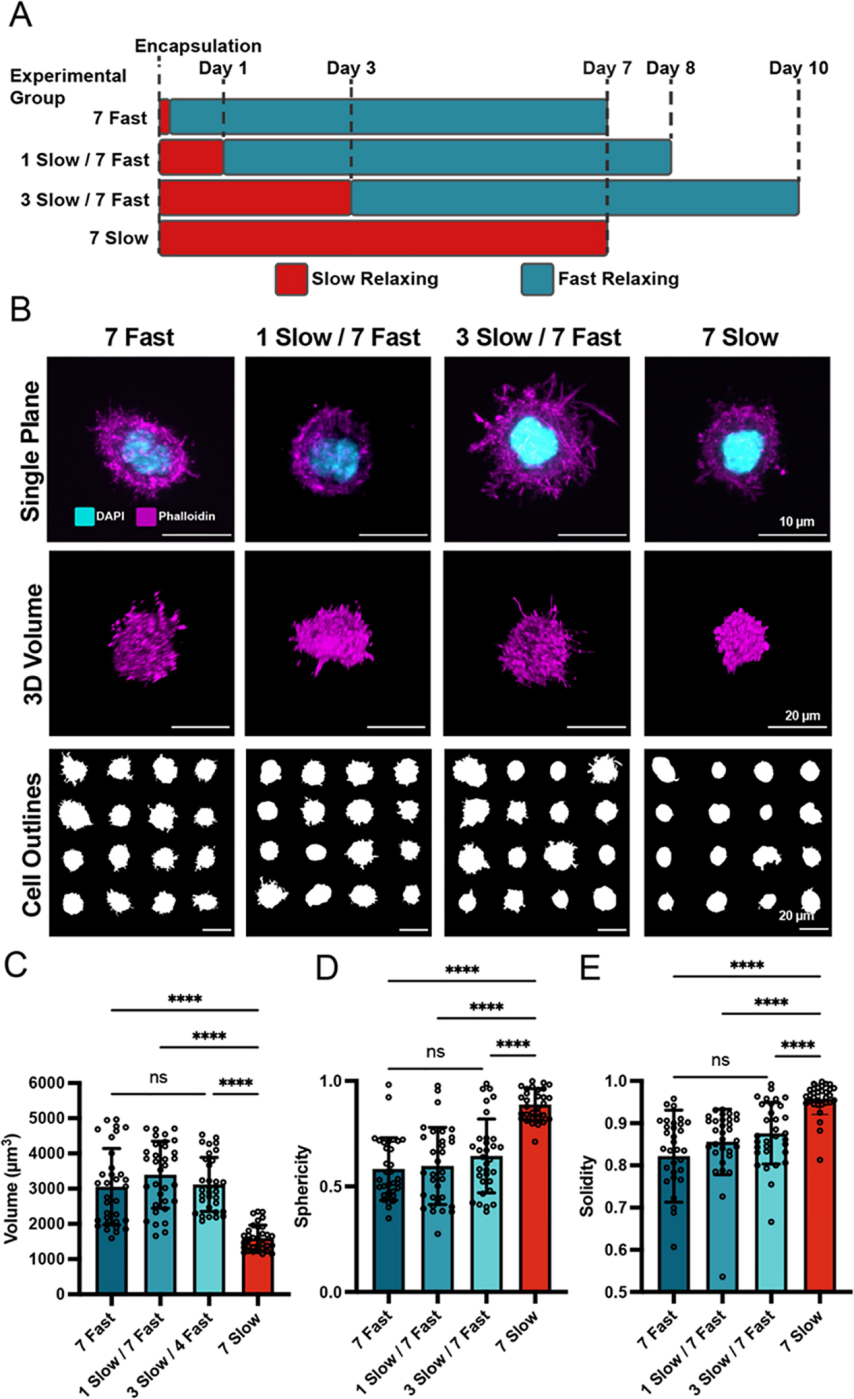
Spread morphologies depend on total time in fast-relaxing conditions, not initial time in slow-relaxing conditions. (a) Experimental timeline to evaluate morphological changes based on initial time in slow-relaxing hydrogels. (b) MSCs exhibit spread morphologies after 7 days in fast-relaxing hydrogels, regardless of initial time in slow-relaxing conditions. Cross-sectional images of single MSCs, volumetric reconstructions of *z*-stacks, and outlines of maximum projections are shown. The cell outlines illustrate a representative sample of the distribution of cell shapes observed in our data set. (c–e) Quantification of shape metrics (volume (c), sphericity (d), and solidity (e)) reveals that cell spreading and protrusions do not significantly differ based on the initial culture period in slow-relaxing hydrogels.

To distinguish the influence of the initial culture period in slow-relaxing matrices from the total time in fast-relaxing matrices, we varied the time in the initial slow-relaxing matrices but maintained the cells in the fast-relaxing matrices for 7 days for all groups (Figure 4A). Between all groups cultured in fast-relaxing conditions for 7 days, we did not observe any significant morphological differences, regardless of the initial time period under slow-relaxing conditions (Figure 4B). Regardless of the day of transition from slow to fast-relaxing matrices, cell spreading was significantly different from cells in slow-relaxing control gels. Cell volumes were similar between all groups in fast-relaxing conditions and higher than slow-relaxing controls (Figure 4C). Similarly, sphericity and solidity were significantly lower in each group that spent 7 days in fast-relaxing conditions (Figure 4D,E). Together, these results indicate that MSC morphology is responsive to the dynamic matrix viscoelasticity. Additionally, cell spreading depends on the time spent in fast-relaxing matrices and is not impeded by initial culture time in slow-relaxing matrices, at least up to 3 days in our experimental design.

### Transition from Slow to Fast-Relaxing Matrices Promotes Proliferation

**Figure 5:**
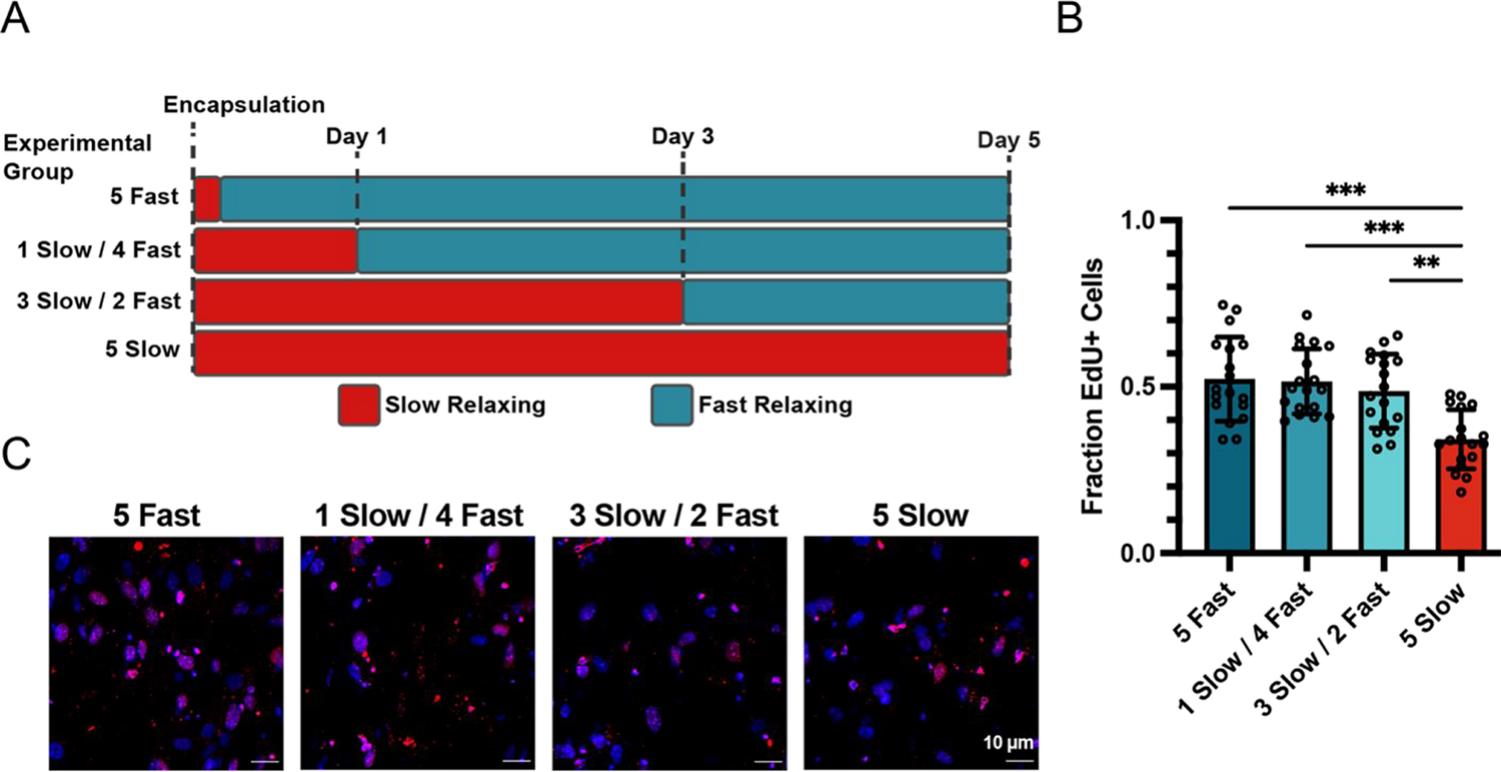
Proliferation rate increases when hydrogel viscoelasticity is dynamically enhanced. (a) Experimental timeline to evaluate proliferation after increasing the stress relaxation rate of hydrogels on days 0, 1, 3, or in unchanged slow-relaxing control conditions. (b, c) MDA-MB-231 cells are significantly more proliferative after dynamically increasing the stress relaxation rate of hydrogels compared to constant, slow-relaxing controls. Proliferation rates do not significantly change based on the day of changing matrix viscoelasticity. EdU staining (red) indicates proliferative cells used for quantification in (b).

We next evaluated the impact of dynamically altering the matrix stress relaxation rate on cell proliferation. Stress relaxation rate was recently shown to regulate proliferation and cell cycle progression in a metastatic breast cancer cell line, MDA-MB-231.^22^ To determine if proliferation rate is also responsive to dynamic changes in matrix viscoelasticity, we cultured MDA-MB-231 cells in matrices that were transitioned from slow to fast relaxation rates at days 0, 1, or 3. All samples were fixed after 5 total days in culture (Figure 5A). Cell proliferation was measured by the incorporation of EdU after 4 days of culture. Regardless of day of matrix transition, fast-relaxing conditions produced a significantly higher number of EdU-positive cells after 5 days compared to cells in slow-relaxing conditions (Figure 5B,C). There were no significant differences in the fraction of EdU-positive cells in matrices that transitioned after 1 or 3 days compared with 5 days in fast-relaxing-only matrices.

Recently, viscoelastic hydrogels have been used to understand the mechanotransduction pathways cells use to sense and respond to varying microenvironmental stress relaxation rates. Integrin clustering and downstream integrin-mediated mechanosignaling were enhanced in faster-relaxing environments, as cells were able to remodel the extracellular environment to bring adhesion ligands into closer proximity.^11,13^ Further, mechanical plasticity of the cellular environment, which is closely coupled to stress relaxation in our work (Figure 2B,C,E), has been shown to enable invadopodial protrusions.^23^ Prior work has also demonstrated that the TRPV4-PI3K/Akt signaling pathway is triggered by fast-relaxing environments to promote cell volume expansion and proliferation.^14,22^ The morphologies adopted by cells in our experiments after matrix stress relaxation are enhanced closely resemble cells from these prior reports, and are suggestive of similar implicated pathways. Our platform will be useful in deciphering the dynamics of these and other stress relaxation-sensitive pathways in future work.

### Spatial Patterning of Viscoelasticity and Effects on Cell Morphology

**Figure 6:**
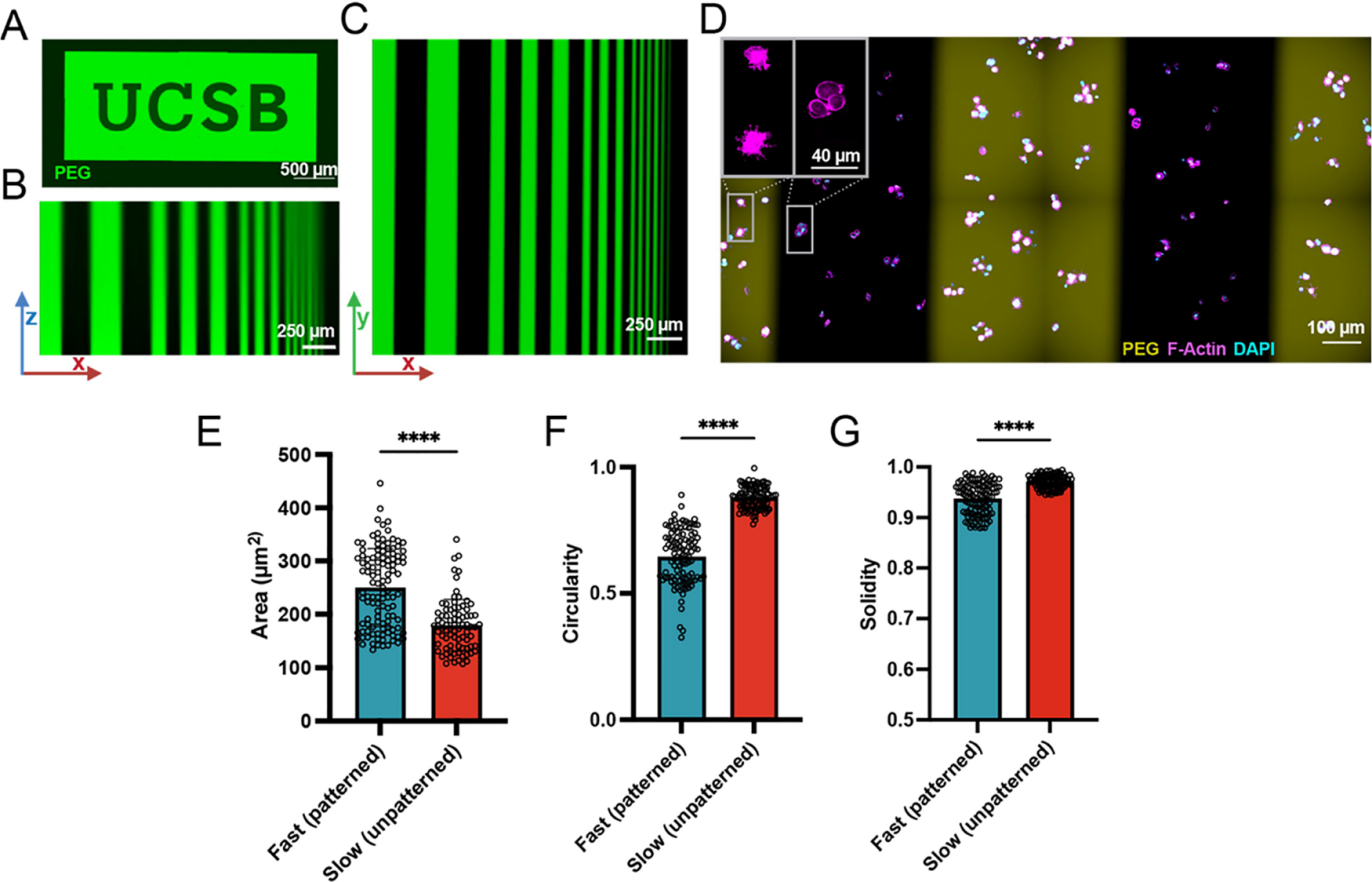
3D hydrogel stress relaxation rate can be spatially photopatterned. (a) Example photopattern to dynamically incorporate fluorescent PEGs to modulate stress relaxation rate locally. (b, c) Demonstration of spatial resolution via photomasking in the *x*–*z* plane (b) and *x*–*y* plane (c). Photopatterning of stress relaxation rates in the presence of MSCs (d). Cells in patterned regions of fast relaxation exhibit more spreading (e), are less rounded (f), and have more protrusions (g).

While photoaddition allows easy modification of alginate stress relaxation properties over time, our approach also enables photopatterning to spatially control cellular behavior through matrix mechanics. To this end, we patterned hydrogels by using a collimated laser source and a laser-printed photomask. To visualize the resulting patterns, 5% v/v of the PEG was labeled with FITC. Patterns could be easily made in a chambered cover glass or glass-bottomed well plate with good fidelity (Figure 6A). To quantify the pattern fidelity in 3D with this system, we patterned the gel with lines of decreasing widths projected through the thickness of the gel (>1 mm). Pattern fidelity in the *x*–*y* plane near the photomask was excellent (Figure 6B,C). Deeper into the gel, 50 μm lines were preserved, but 25 μm lines became distorted. Notably, the spatial resolution we achieved is still sufficient for all but patterning at the scale of single cells deep into hydrogels. Further, this limitation is a result of the optical setup and could be overcome with more advanced photopatterning techniques that have been previously utilized with thiol–ene photochemistry.^47,48^ How closely the resolution of mechanical changes mirrors that of the photocoupling of PEG is not clear, and this may limit the use of this approach for very fine mechanical changes (i.e., subcellular features).

To demonstrate the utility of this capability, we encapsulated MSCs in a uniformly slow-relaxing 3D gel and then patterned 250 μm lines of fluorescent PEG to enhance stress relaxation. After 7 days of culture in the patterned gel, the cells were fixed and stained with phalloidin and DAPI. Using a tiled scan of whole gels to unbiasedly image the samples, different morphologies were observed in regions with and without PEG, indicating cellular responses to local viscoelasticity differences. (Figure 6D). Cell morphologies in regions with and without PEG differed in area, circularity, and solidity (Figure 6E–G). Cells in PEG patterned regions showed greater areas and decreased circularity and solidity, indicative of their greater number and larger size of protrusions. Overall, we demonstrate that this system can be used to pattern gel mechanics spatially and that cells have a similar morphological response to being in a fast-relaxing local region of a gel as they do to being in an entirely fast-relaxing gel.

We anticipate that 3D cell culture platforms with spatiotemporally tunable stress relaxation rates will have broad utility in a number of applications. The ECM of developing tissues is highly dynamic, and tunable systems could be used to pattern or direct morphogenetic processes such as organoid maturation, symmetry breaking, or crypt formation,^21^ glandular branching, or neovascularization.^15^ Fibrotic progression is marked by remodeling of the ECM over time that results in substantial mechanical changes. How stress relaxation rates are altered in a fibrotic microenvironment is still not well characterized, but dynamically tunable platforms will enable modeling of the viscoelasticity during fibrotic progression or resolution to understand the impact on cell behavior. In addition, there are many outstanding and fundamental questions in the field of mechanobiology that dynamic platforms could be used to address. For example, investigating the extent and basis of mechanical memory in response to changing stress relaxation rates and the effect of viscoelastic gradients on cell migration, particularly in 3D environments. We expect the impact and utility of these platforms to grow as they are introduced to the broader field of researchers performing 3D cell culture.

## Conclusions

We developed a method to increase the stress relaxation rate of the 3D hydrogels in the presence of cells. Our light-triggered approach can modulate stress relaxation rates over the range in which cells sense and respond to matrix viscoelasticity. We found that cell protrusions, spreading, and shape are responsive to dynamic changes in the matrix stress relaxation rate. Additionally, the cell proliferation rate is also sensitive to changes in matrix viscoelasticity. We utilized the light-based approach to show high spatial control of 3D viscoelasticity by photopatterning as well, and we again demonstrated the morphological response of cells in distinct viscoelastic environments. This platform addresses a critical unmet need for modeling spatiotemporally dynamic cellular microenvironments with a 3D in vitro cell culture. We also envision this platform in applications for guiding or directing cell fate and tissue geometry over time and time.
